# The Novel Herbal Cocktail AGA Alleviates Oral Cancer through Inducing Apoptosis, Inhibited Migration and Promotion of Cell Cycle Arrest at SubG1 Phase

**DOI:** 10.3390/cancers12113214

**Published:** 2020-10-31

**Authors:** Jui-Hua Lu, Yen-Ru Chou, Yue-Hua Deng, Mao-Suan Huang, Shaw-Ting Chien, Bach Thi Nhu Quynh, Chia-Yu Wu, Edlin Anahi Peláez Achtmann, Hsin-Chung Cheng, Navneet Kumar Dubey, Win-Ping Deng

**Affiliations:** 1 School of Dentistry, College of Oral Medicine, Taipei Medical University, Taipei 11031, Taiwan; d225101001@tmu.edu.tw (J.-H.L.); m204108001@tmu.edu.tw (Y.-H.D.); m204107011@tmu.edu.tw (E.A.P.A.); g4808@tmu.edu.tw (H.-C.C.); 2 Stem Cell Research Center, College of Oral Medicine, Taipei Medical University, Taipei 11031, Taiwan; chiens3@uw.edu; 3 Graduate Institute of Biomedical Materials and Tissue Engineering, College of Biomedical Engineering, Taipei Medical University, Taipei 11031, Taiwan; d225102008@tmu.edu.tw; 4 Department of Dentistry, Taipei Medical University-Shuang Ho Hospital, New Taipei City 235, Taiwan; 08686@s.tmu.edu.tw; 5 Department of Medicine Molecular Biology, Haiphong University of Medicine and Pharmacy, Dang Giang, Ngo Quyen, Haiphong 04212, Vietnam; btnquynh@hpmu.edu.vn; 6 Division of Oral and Maxillofacial Surgery, Department of Dentistry, Taipei Medical University Hospital, Taipei 11031, Taiwan; m204097001@tmu.edu.tw; 7 School of Dental Technology, College of Oral Medicine, Taipei Medical University, Taipei 11031, Taiwan; 8 Department of Dentistry, Taipei Medical University Hospital, Taipei 110131, Taiwan; 9 Graduate Institute of Biomedical and Pharmaceutical Science, Fu Jen Catholic University, Taipei 242, Taiwan; 10 Department of Life Science, Tunghai University, Taichung 407224, Taiwan

**Keywords:** Antler’s extract, *Ganoderma lucidum*, *Antrodia Camphorata*, oral cancer, anti-oxidation, cell cycle

## Abstract

**Simple Summary:**

Oral cancer is a major cause of death from oral disorders worldwide. These are highly lethal, incapacitating and present mostly in the form of squamous cell carcinomas. Owing to therapy resistance, the impact of conventional drugs is limited. Therefore, we aimed to develop a novel anti-oral cancer therapeutic alternative in the form of our synthesized robust cocktail of Antler’s extract (A) and *Ganoderma lucidum* (G) and *Antrodia Camphorata* (A), designated as AGA. We demonstrated that AGA, primarily contained with bioactive components such as triterpenoids and polysaccharides strongly, induces cellular apoptosis and inhibits cell migration and cyclin expression, leading to promoted cell cycle arrest at the subG1 phase. AGA could also efficaciously retard tumor growth without any toxic influence on liver and kidneys. Therefore, AGA holds a great potential to be an oral cancer treatment strategy.

**Abstract:**

Traditional Chinese medicines Antler’s extract (A) and *Ganoderma lucidum* (G) and *Antrodia Camphorata* (A) have been known to individually contain a plethora of bioactive factors including triterpenoids, polysaccharides etc., exerting various curative impacts such as anti-inflammatory, anti-oxidative, anti-atherosclerotic and anti-viral activities. However, their combinatorial therapeutic efficacy for oral cancer has not been investigated. Hence, we synthesized a robust cocktail called AGA and investigated its anti-oral cancer potential in vitro and in vivo. An MTT assay revealed the IC_50_ of AGA to be about 15 mg at 72 h. Therefore, 10 mg and 20 mg doses were selected to study the effect of AGA. The AGA significantly inhibited proliferation of oral cancer cells (HSC3, SAS, and OECM-1) in a dose- and time-dependent manner. AGA retarded cell cycle regulators (CDK4, CDK6, cyclin A, B1, D1 and E2) and apoptosis inhibitory protein Bcl-2, but enhanced pro-apoptotic protein Bax and a higher percentage of cells in Sub-G1 phase. Mechanistically, AGA suppressed all EMT markers; consequently, it decreased the migration ability of cancer cells. AGA significantly reduced xenograft tumor growth in nude mice with no adverse events in liver and renal toxicity. Conclusively, AGA strongly inhibited oral cancer through inducing apoptosis and inhibiting the migration and promotion of cell cycle arrest at subG1 phase, which may be mediated primarily via cocktail-contained triterpenoids and polysaccharides.

## 1. Introduction

Oral cancer is one of the malignant tumors that affects human health and life worldwide, and ranks fifth among the top ten causes of cancer-associated deaths, and since 2003, it has been the fourth most common cancer among males for 12 consecutive years in Taiwan [1]. Currently, apart from the surgical approach, chemotherapy and radiotherapy are the major therapeutic strategies for oral cancer. However, these strategies have been known to cause adverse effects including nausea, diarrhea, poor quality of life and others. Hence, the development of novel anti-oral cancer therapeutic agents is urgently required. Recent years have witnessed concerted efforts to explore the valuable biofactory of traditional Chinese medicines (TCM) as therapeutic alternatives, owing to their small side effects [2].

Based on our previous study on the cytotoxic effects of *Antrodia camphorata* [3], we synthesized a robust cocktail of three TCM, including Antler’s extract (A) and *Ganoderma lucidum* (G) and *Antrodia Camphorata* (A), which was designated as AGA. Velvet antlers have been demonstrated to contain polysaccharides, proteins, amino acids, polypeptides, mineral elements and fatty acids [4]. Reports have evidenced that antlers exhibit various therapeutic activities in physical fatigue, osteoporosis, hypercholesterolemia and myocardial infarction, wound healing and rheumatoid arthritis [5,6]. Multifunctional peptides of velvet antlers have gained much attention in the area of food science owing to their low toxicity and their rapid intestinal absorption and therapeutic potential [4]. Antlers possess immunomodulatory activities, which have been evidenced to increase monocytes, indicating an immune system enhancing function [7].

Furthermore, the fruiting bodies of *Ganoderma lucidum* has been reported to contain a plethora of bioactive compounds, including triterpenoids, polysaccharides, steroids, fatty acids, nucleotides, sterols and peptides, imparting numerous medicinal impacts such as anti-inflammatory, anti-oxidative, anti-atherosclerotic, anti-viral, anti-microbial, anti-tumor, hypolipidemic, anti-diabetic and anti-fungal activities [8]. Of these, the most pharmacologically active compounds include triterpenoids and polysaccharides.

*Antrodia camphorata* is a unique mushroom of Taiwan, which has been used as a traditional medicine for the protection of diverse health-related conditions through synergistic effects of its pharmacologically active components including steroids, triterpenoids, polysaccharides, lignans, phenyl derivatives, fatty acids and the trace elements contained in fruiting bodies and mycelium [3,9]. These components render it a potent direct-free radical scavenger. Therefore, we synthesized a robust cocktail of these TCMs and aimed to investigate its anti-oral cancer potential in vitro as well as in vivo. Additionally, the potential molecular mechanisms underlying these activities of AGA was also explored by determining the expression levels of regulating key genes and proteins involved in apoptosis, migration and cell cycle arrest.

## 2. Results

### 2.1. AGA Extract Inhibits Oral Cancer Cell Viability and Colony-Forming Ability

**Figure 1:**
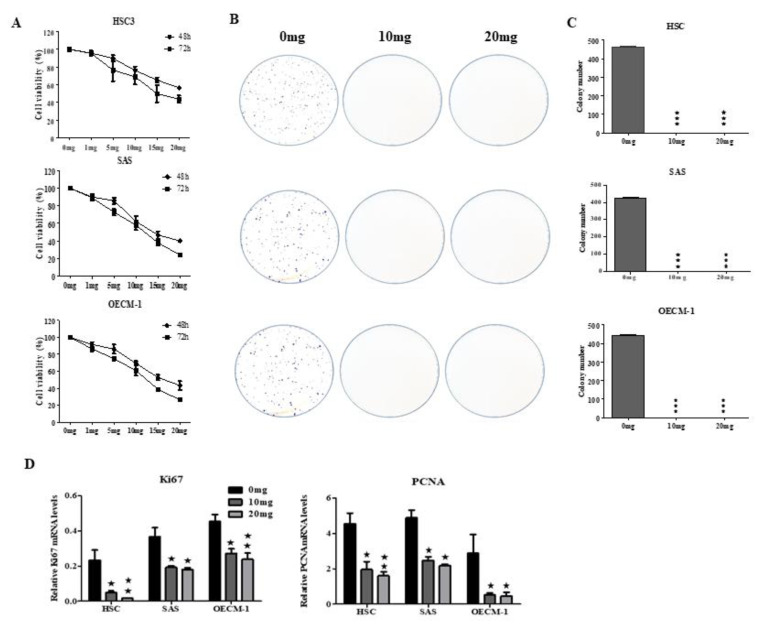
The cytotoxic effect of Antler’s extract, *Ganoderma lucidum* and *Antrodia Camphorata* (AGA) extract (0–20 mg/mL) on human oral squamous cell carcinoma cells (HSC3), human tongue squamous carcinoma (SAS) and oral epidermoid carcinoma cell, Meng-1 (OECM-1). (**A**) AGA dose optimization for cell viability revealing IC_50_ value of about 15 mg. Thereafter, the dose of AGA was selected as 10 and 20 mg/mL and their effect on the (**B**) Colony-forming ability of oral cancer cells was determined for 72 h, which was later relatively quantified. (**C**) Relative expressions of Ki67 and proliferating Cell Nuclear Antigen (PCNA) were detected by Western blot (**D**). Data are represented in triplicates as mean ± SD. * *p* < 0.05, ** *p* < 0.01, and *** *p* < 0.001 compared to control.

The cell viability of oral cancer cells was evaluated by MTT assay on HSC3, SAS and OECM-1 cells lines treated with AGA extract with different concentrations (0–20 mg/mL) for 48 h and 72 h. Our result showed that AGA extract exerted an inhibitory effect on cell viability of all oral cancer cells in a concentration-dependent manner with an IC50 value of about 15 mg at 72 h (Figure 1A). Therefore, we selected 10 mg and 20 mg doses and a time point of 72 h to AGA extract for further studies.

To explore the role of AGA extract on proliferation of HSC3, SAS and OECM-1 cells, a colony-forming assay was performed. As revealed in Figure 1B, compared to control, the abilities of forming colonies were significantly diminished by AGA extract in a concentration-dependent fashion. These results are also in line with their relative quantification of a number of colonies formed (Figure 1C). We further explored the inhibitory effect of AGA extracts on the proliferation effect of oral cancer lines, by detecting the expression levels of Ki-67 antigens (Ki-67) and proliferating Cell Nuclear Antigens (PCNAs), the classic markers of cellular proliferation [10]. These biomarkers were also found to be significantly inhibited in a concentration-dependent manner (Figure 1D).

### 2.2. AGA Extract Impacts the Expression Levels of Cell Cycle and Apoptosis Regulators in Oral Cancer Cells

**Figure 2:**
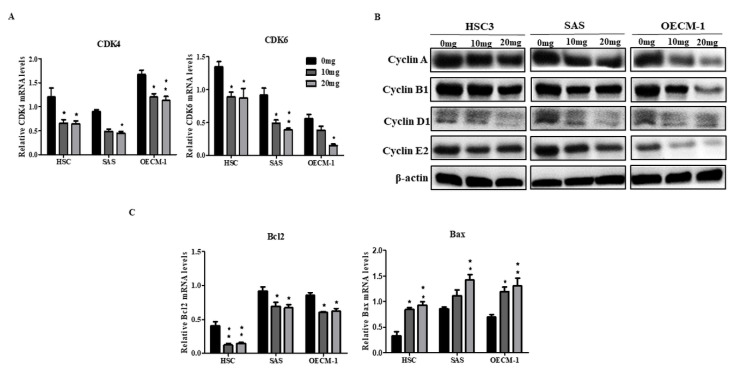
Impact of AGA on expression cell cycle-related factors in ) oral cancer cells (HSC3, SAS and OECM-1). (**A**) qPCR-dependent mRNA levels of CDK4 and CDK6. (**B**) Cyclins A, B1, D1 and E2 were determined by Western blot. (**C**) mRNA levels of apoptosis biomarkers, Bcl-2 and Bax. Data are represented in triplicates as mean ± SD. * *p* < 0.05 and ** *p* < 0.01 compared to control.

After observing the inhibitory effect of AGA extracts on the viability and colony formation ability of oral cancer cells, we further evaluated by the expression levels of cell cycle regulators (CDK4, CDK6, cyclin A, B1, D1 and E2) by qPCR and Western blot. After 72 h, the mRNA levels of CDK4 and CDK6 were significantly decreased (Figure 2A). Similarly, protein levels of cyclin A, B1, D1 and E2 were also inhibited (Figure 2B). Furthermore, as the Bcl-2 and Bax proteins play a key role in the regulation of apoptosis, we examined the effects of AGA extract on the expression of these regulatory factors. Our data showed that AGA extract significantly decreased the expression of the apoptosis inhibitory protein Bcl-2, whereas it increased the expression of the pro-apoptotic protein Bax (Figure 2C).

### 2.3. AGA Extract Influence the Phase of Cell Cycle Progression and Apoptosis in Oral Cancer Cells

**Figure 3:**
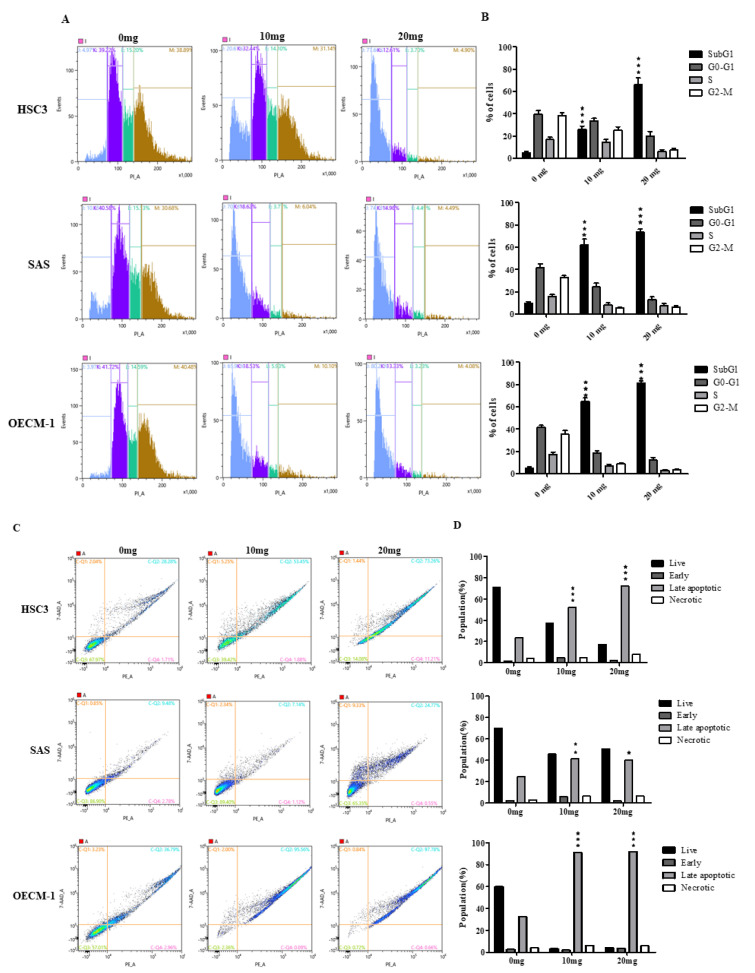
Effects of AGA on the phases of cell cycle distribution and apoptosis in oral cancer cells (HSC3, SAS and OECM-1). (**A**) Representative flow cytometric histograms cell cycle phases (Sub G1, G0–G1, S and G2-M) and their relative percentage population (**B**). Histogram revealing the live, early, late and necrotic phase of apoptosis of oral cancer cells after their treatment with AGA extract (**C**) and their relatively quantified population (**D**). Data are represented in triplicates as mean ± SD. * *p* < 0.05, ** *p* < 0.01 and *** *p* < 0.001 compared to control.

We next determined the cell cycle phases of oral cancer cells after their treatment with AGA extract for 72 h. The flow cytometric analysis-based histogram revealed a higher percentage of cells in the Sub-G1 phase (apoptotic cells), particularly in SAS and OECM-1 cells (Figure 3A), indicating an increased number of apoptotic cells. These results are also in line with their relatively quantified population (Figure 3B), both at 10 as well as 20 mg concentrations. We further assessed the rescuing ability of AGA on oral cancer by determining the specific phases of apoptosis, which clearly revealed a higher percentage of oral cancer cells, specifically HSC3 and OECM in the late apoptosis phase (Figure 3C), which were also confirmed through their relative quantification (Figure 3D).

### 2.4. AGA Extract Inhibits Migration in Oral Cancer Cells

**Figure 4:**
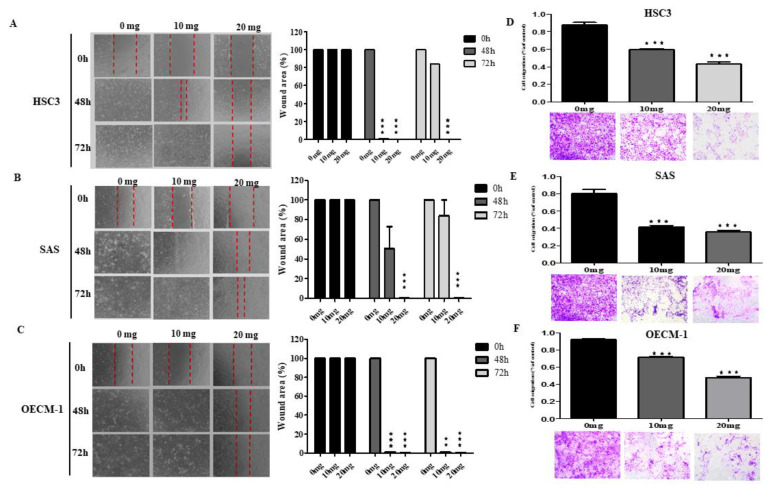
Efficacy of AGA extract on cell migration in oral cancer cells. Cell motility of HSC3, SAS and OECM-1 upon AGA treatment was determined by wound-healing assay at 0, 10 and 20 mg/mL for 48 h and 72 h. Representative photomicrographs (magnification, 100 µm) of wound healing in (**A**) HSC3, (**B**) SAS and (**C**) OECM-1 cells with their relatively quantified wound area (%). Transwell assay-dependent analysis of invasive ability of (**D**) HSC3, (**E**) SAS and (**F**) OECM-1 cells (magnification, ×100), and their relative qualification. Data are represented in triplicates as mean ± SD. ** *p* < 0.01 and *** *p* < 0.001 compared to control (0 mg).

To explore whether the anticancer effect of the AGA extract in vitro is associated with cell migration, we examined the motility of oral cancer cells via the scratch wound healing assay. Cells with 90% confluence were scratched to create the wounds and were then treated with a various concentration of AGA extract (0–20 mg/mL) and wound-healing was observed at 48 h and 72 h. Our in vitro results implied a significant delaying of AGA-treated oral cancer cell motility in HSC3, SAS and OECM-1 cells (Figure 4A–C, respectively) in a dose- and time-dependent manner, which has also been confirmed with their relatively quantified wound area (%). Consistent with the wound healing assay, the transwell assay indicating cellular mobility also confirmed that the AGA extract treatment resulted in a marked decrease in the migration ability of HSC3, SAS and OECM-1 cells (Figure 4D–F, respectively) through the porous membrane (as stained purple) in a dose- and time-dependent manner. These results were also found to be in concord with their relative quantification.

### 2.5. AGA Extract Influence EMT and its Regulatory Factors in Oral Cancer Cells

**Figure 5:**
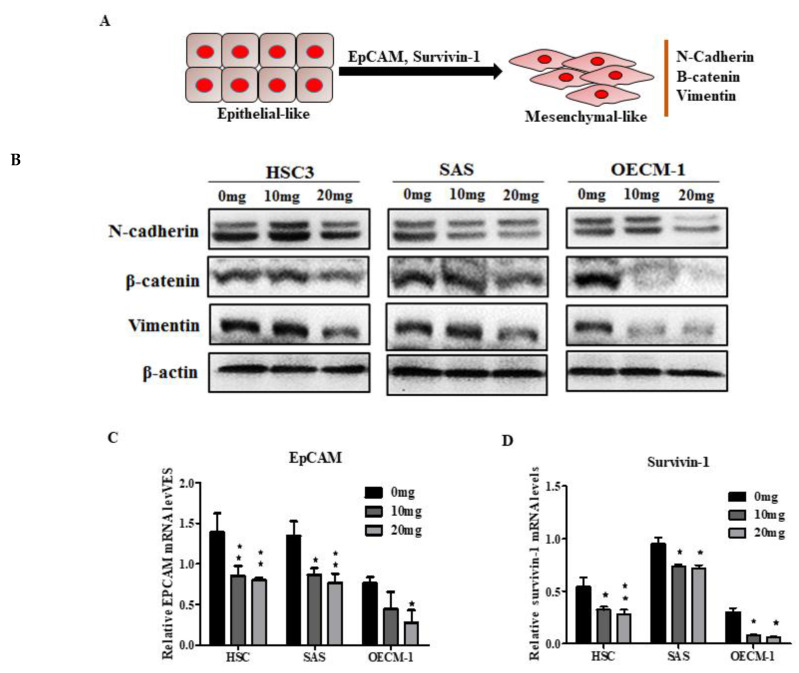
Influence of AGA on epithelial mesenchymal transition (EMT) markers. (**A**) Schematic representation of EMT showing increased motility of oral cancer cells and enabling them to develop into an invasive phenotype, (**B**) N-cadherin, β-catenin and vimentin, and its regulatory factors, i.e., (**C**) EpCAM and (**D**) survivin-1 in HSC3, SAS and OECM-1 oral cancer cells. * *p* < 0.05 and ** *p* < 0.01 compared to control (0 mg).

To investigate the mechanism by which AGA extract treatment inhibited the migration and wound healing, we examined whether it could affect EMT markers (N-cadherin, β-catenin and vimentin), representing the increased motility of oral cells and enabling them to develop into an invasive phenotype (Figure 5A). Our Western blot results revealed a markedly inhibited expression of all the EMT markers, particularly at a high dose (20 mg) (Figure 5B). Furthermore, we investigated the expression levels of epithelial cell adhesion molecules (EpCAM) (Figure 5C) and survivin-1 (Figure 5D), which are promoters of EMT leading to cancer cell migration and invasion [11,12]. In consistence with EMT results, the qPCR data also demonstrated significantly suppressed levels of expression of EpCAM and sirvivin-1, indicating the EMT-inhibiting characteristics of AGA.

### 2.6. Inhibitory Effect of AGA on Tumorigenesis in Nude Mice

**Figure 6:**
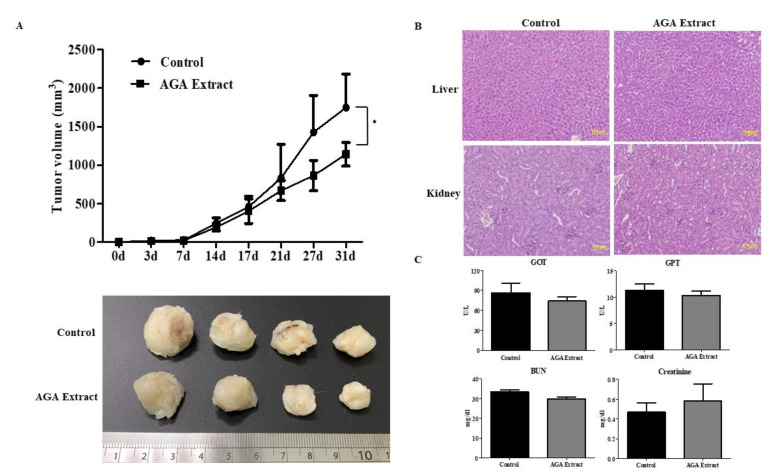
Impact of AGA on tumorigenesis and organ toxicity. (**A**) Representative photomicrographs of tumors and its quantified volume derived from mice with oral cancer. (**B**) AGA-associated hepatic and renal toxicity was assessed through hematoxylin and eosin-stained sections of liver and kidney. Magnification, 20X (scale: 100µm). (**C**) Blood serum levels of glutamate oxaloacetate (GOT) and glutamate pyruvate transaminase (GPT) represents liver function, whereas blood urea nitrogen (BUN) and creatinine indicate renal function. Data are represented in triplicates as mean ± SD, * <0.05 (Oral cancer (Control) = 5, AGA-Oral cancer = 5).

To assess the in vivo antitumor effects of AGA extract on the initiation and progression of oral cancer, we examined the rate of tumor growth and tumor volume in nude mice upon administration of AGA extract. The tumors were excised surgically at the time of sacrifice 31 (days). We observed reduced tumor size in the AGA-treated group, when compared to the oral cancer group (control) (Figure 6A, lower panel), which was further confirmed through the significantly suppressed tumor volume (Figure 6A, upper panel). We further monitored the possibility of adverse events in terms of liver and renal toxicity. Firstly, AGA-associated hepatic and renal toxicity was assessed through hematoxylin and eosin-stained sections of liver and kidney. The results showed no change in morphology of the control and AGA-administered groups (Figure 6B). Specifically, no significant difference between blood serum levels of GOT and GPT representing liver function was exhibited (Figure 6C, upper panel). Beside, BUN and creatinine indicating renal function also remained unchanged in both groups (Figure 6C, lower panel). Taken together, these results exclude the possibility of adverse events by AGA therapy.

## 3. Discussion

Oral cancer is a genetically complex and very aggressive disorder [13]. Apart from the development of secondary and primary tumors, tumor invasion, high rates of locoregional recurrence and lymph node metastasis are the major causes of death among oral cancer patients. Furthermore, the survival rates of these patients are in the range of 40 to 50%, which has not significantly changed over the past few decades [14]. Since cancer patients treated by chemotherapy and/or radiotherapy often exhibit deleterious side-effects, the traditional Chinese medicine in cancer prevention and therapy has received great attention as an efficacious alternative. In South East Asia, the Antler’s extract (A) and *Ganoderma lucidum* (G) and *Antrodia Camphorata* (A) have been individually used as a home remedy in traditional Chinese medicine (TCM) for a long time. To date, a few studies have demonstrated the presence of mineral elements, polypeptides, proteins, amino acids, polysaccharides and fatty acids in velvet antlers [4]. Being indigenous subspecies of Taiwan, the Formosan sambar deer (*Cervus unicolor swinhoei*) and Formosan sika deer (*Cervus nippon taiouanus*) have been the prominent source of antlers. Studies have also revealed that velvet antlers have various pharmacological impacts, including tissue repair, wound healing, and rheumatoid arthritis [5]. In a seminal study by Xiao et al. [15]. velvet antlers inhibited ischemia-hypoxia cardiac microvascular endothelial cell injuries through regulating the PI3K/Akt signaling pathway. Antlers also exhibited anti-fatigue effects such as GnRH signaling pathways and insulin signaling pathways [6].

Besides, the medicinal mushroom *G. lucidum*, has been reported to possess anti-inflammatory activities, analgesic effects, antitumor activities and anti-HIV-1 activities [16,17,18,19]. The isolated *G. lucidum* fruiting bodies, mycelia and spores contain bio-active substances such as triterpenoids, polysaccharides, amino acids, peptides, fatty acids, oligosaccharides and other trace elements [20]. *Antrodia camphorata* is a traditional herbal medicine from Taiwan, which belongs to the polyporaceae Basidiomycota family and contains tremendous pharmacologically active components including triterpenoids, polysaccharides, steroids, lignans, phenyl derivatives, fatty acids and trace elements [3]. A protective impact of arginine consumption against oral cancer is biologically plausible, which may be mediated by the ability of arginine to reduce cell proliferation and ornithine decarboxylase activity [21]. In addition to this, the gluconate may be partially attributed to an increased anti-cancer impact when combined with other therapeutics, such as Zn2+ [22]. Furthermore, maltodextrin also imparts tumor suppression by inducing apoptosis via phosphorylation of Akt [23]. Based on our therapeutics evidenced on *antrodia camphorata*, we synthesized a robust cocktail, i.e., AGA, in addition to minor quantities of L-arginine, Zinc gluconate and maltodextrin, to investigate its anti-oral cancer potential.

Our study revealed that AGA significantly suppressed HSC3, SAS, and OECM-1 cell proliferation in a dose- and time-dependent fashion. This was further confirmed through expression levels of proliferating cell nuclear antigen (PCNA), an intranuclear polypeptide synthesized only in proliferating cells [24]. Therefore, the PCNA is a biomarker of cell proliferation activity, which is associated with cell cycle, particularly in S, G1 and G2 phases. Furthermore, Ki-67 is a type of nuclear antigen which is expressed in G1, S, G2, and M phases of cell cycle [25,26]. Previous studies have demonstrated the high expression of Ki-67 in the liver, prostate, gastric and other multiple tumor cells. Collectively, the higher expression intensity of PCNA and Ki-67 indicating the proliferation activity and malignancy of oral cancer cells was dramatically decreased by AGA treatment. Furthermore, as the progression of the cell cycle is regulated by a complex of cellular cyclins and cyclin-dependent kinases (CDKs) [27], our results suggest that the anti-proliferative effects of AGA in oral cancer cells may be through inhibiting CDK4 and CDK6. In a previous study by Ruan et al., a mixture of triterpenoids of *G. lucidum* induced cell accumulation at the G1 phase in Hela cells [28]. Another study also revealed that *G. lucidum* triterpenoid induced mainly cell cycle arrest at the G1/G0 phase, which was due to the upregulation of p21 expression and the downregulation of CDK4 expression in prostate cancer cells [29]. These evidences strongly support our findings that a higher amount of triterpenoid present in our AGA cocktail may contribute in cell cycle arrest. Therefore, this could be one of the potential mechanisms through which AGA may impart its anti-proliferation activity in oral cancer cells.

Furthermore, apoptosis is cell death and defense mechanisms precisely regulate cell numbers to remove unwanted and potentially hazardous cells [30]. During tumorigenesis, the deregulation of the biochemical pathways control apoptosis as well as the deregulation of cell proliferation. The Bcl-2 protein is localized in the outer mitochondrial membranes and various reports imply that it may block apoptosis by inhibiting the release of cytochrome c from mitochondria. Many studies have also reported that Bcl-2 is highly expressed in the cancerous state, and enhanced expression of Bax increases apoptosis [31,32]. In the present study, we found that AGA was effective in inducing apoptosis in oral cancer cells in a dose-dependent manner, which was revealed by their higher population in the sub G1 state of the late apoptotic phase.

Additionally, we demonstrated that AGA retarded cellular migration as revealed by wound healing and transwell assays. This effect is promoted my EMT programs which alter the cell state and invasive behavior, and could be evidenced with significant upregulation of N-cadherin, β-catenin and vimentin [33]. These are essential proteins of EMT, which contribute to cancer invasion and tumor metastasis and are characterized by upregulated mesenchymal markers. A study showed that N-cadherin induces EMT and cancer stem cell-like characteristics through activating the ErbB pathway by upregulating ERK, growth factor receptor-bound protein 2 (GRB2), and SHC-transforming protein [34]. This study further revealed that in prostate cancer cells overexpressed with N-cadherin, increased levels of pluripotency-associated markers have also been evidenced. Furthermore, AGA also inhibited EMT promoters, the EpCAM and survivin-1, [11,12], indicating its EMT-inhibiting characteristics.

To further examine the anti-tumorigenic impact of AGA in oral cancer, we conducted an in vivo study using xenograft nude mice. We observed that AGA could significantly inhibit the formation and growth of xenograft tumors in nude mice. In line with our study on *Antrodia camphorata* [3], a previous study has shown that methanol extract from *G. lucidum* could significantly inhibit B16 mouse melanoma growth in vivo [35]. Furthermore, the results of Hsieh et al. implied that *A. camphorata* could act as a non-toxic, antioxidant, antimutagenic and DNA-protective agent and could be developed into health foods [36]. In a similar trend, Antrodin A contained in *Antrodia camphorata* has been attributed to improve the antioxidant and anti-inflammatory capacities of the liver and maintain the stability of intestinal flora [37]. Based on these evidences, it could be anticipated that our AGA cocktail could be a stronger anti-tumor agent compared to individual bioactive components. In a seminal report, the ethanol extract of *G. lucidum* effectively rescued ethanol-induced acute hepatic injury in SD rats through regulating the activities of ethanol-metabolizing enzymes and by inhibiting oxidative stress [38]. Furthermore, a study by Shi et al. demonstrated that mice treated with *G. lucidum* peptides suppressed D-galactosamine (D-GalN)-induced hepatic injury, along with a significant reduction in the activity of superoxide dismutase (SOD) and glutathione (GSH) levels in the liver [39]. However, we found that AGA supplementation showed no effect on the structure and function of liver and kidneys, as shown by their morphology and the biochemical profile of control and AGA-administered groups, indicating its safety.

## 4. Materials and Methods

### 4.1. Cell Lines and Reagents

The oral squamous cell carcinoma (OSSC) cell line was provided by Cheng-Chieh Yang, Institute of Oral Biology, Department of Stomatology, Taipei Veterans General Hospital, Taipei, Taiwan. The SAS and OECM-1 cells were grown in Dulbecco’s modified Eagles medium (DMEM; Gibco/Life Technologies, Waltham, MA, USA), and HSC-3 cells were in DMEM/F12 medium (Gibco/Life Technologies) with 10% fetal bovine serum (FBS; Corning; Merck, Kenilworth, NJ, USA, KGaA) and 1% penicillin/streptomycin (Sigma-Aldrich; Merck KGaA) and maintained in a humidified 37 °C incubator with 5% CO2.

### 4.2. Preparation of the Herbal Cocktail AGA

The robust cocktail contains mainly the Traditional Chinese medicines Antler’s extract (A) and *Ganoderma lucidum* (G) and *Antrodia Camphorata* (A), in addition to minor quantities of L-arginine, Zinc gluconate and maltodextrin, which were provided by Well Shine Biotechnology Development Co., Ltd., Taipei, Taiwan.

### 4.3. Cell Viability Assay

HSC-3, SAS and OECM-1, oral cancer cells were seeded in 96-well micro titer plates in triplicate at a density of 2000 cells per well and were grown overnight at 37 °C. The next day, the cells were treated with AGA extract with (0–20 mg/mL) incubated for 48 h and 72 h. Then, the MTT assay-dependent cell viability was determined by adding 25 μL methylthiazolyldiphenyl-tetrazolium bromide (Sigma-Aldrich, St. Louis, CA, USA, Cat No. M5655) to each well and incubated for 4 h, followed by the addition of 150 μL of solubilization solution N, N-dimethylsulfoxide (DMSO) (Sigma-Aldrich, Cat. No. D5879). The plates were placed on a shaker at room temperature overnight to allow complete lysis of the cells and were read at 595 nm the following day. Experiments and data processing were performed as described previously. Half-maximal inhibitory concentrations (IC50) were determined using Sigma Plot 12.0 Software (Systat Software, Inc., San Jose, CA, USA). Combination index (CI) was performed using data obtained from an MTT assay with CompuSyn software. The CI values indicate a synergistic effect when <1, an antagonistic effect when >1, and an additive effect when equal to 1.

### 4.4. Colony Formation Assay

HSC-3, SAS and OECM-1, oral cancer cells were seeded (500 cells/well) in 10 mm cell culture dishes and incubated at 37 °C overnight. The following day, cells were incubated with AGA extract (0, 10 and 20 mg/mL) and incubated for 2 weeks. The culture medium was changed in 3 days with fresh medium, and the medium was changed every week for another 2 weeks. Then, cells were washed twice with PBS, fixed with cold methanol for 30 min at 4 °C and stained with crystal violet dye (0.1% *w/v*) at room temperature for 1 h. The plates were washed with water, dried and scanned.

### 4.5. Wound-Healing Assay

HSC-3, SAS and OECM-1, oral cancer cells were seeded (1.5 × 10^5^ cells/well) in six-well plates and incubated at 37 °C overnight. Next day, when cells reached 100% confluence, a straight line with the same width was scratched across the monolayer through a 100-μL pipette tip. After PBS washing to remove non-adherent cells, cells were further treated with AGA extract with (0, 10 and 20 mg/mL) incubated for 48 h and 72 h, and 3 pictures of randomly selected fields at the lesion border were acquired under an Olympus IX-71 (Olympus Opticol Co., Tokyo, Japan) inverted microscope.

### 4.6. Transwell Migration and Invasion Assays

In vitro cell migration was examined using the 8 μm BD Falcon cell culture insert (BD Biosciences, NJ). SAS, OECM-1, HSC-3 oral cancer cells were seeded (1 × 10^5^ cells/well) were suspended in 500 μL of serum-free DMEM and then further seeded into the upper compartment of each chamber. The lower compartment was filled with 1 mL of DMEM containing 10% FCS. After 72 h of incubation at 37 °C in 5% CO2, the non-migrating cells were removed by scraping the upper surface of the membrane. Cells on the reverse side were stained with 0.1% crystal violet, and migrating cells were counted under a microscope (Olympus IX71, Tokyo, Japan). Thereafter, the through the MTT assay, we determined whether the effects of AGA extract (0,10 and 20 mg/mL) on cell migration were due to the inhibition of cell viability.

### 4.7. Cell-Cycle Analysis

For cell-cycle analysis by flow cytometry, the cells were trypsinized and washed with PBS and fixed with 75% ethanol. Then, 500 µL of RNase A (0.2 mg/mL, Sigma-Aldrich, 10109142001) and 500 µL of propidium iodide (0.02 mg/mL, Sigma-Aldrich, 11348639001) were added to the cell suspensions, and the mixtures were incubated for 30 min in the dark. A flow cytometer (BD FACS Calibur) was used for cell-cycle analysis, and 10,000 events for each sample were recorded. Data acquisition and analysis were done using BD FACSDiva software version 4.1 (BD Biosciences, San Jose, CA, USA), and the percentages of cells present in the G1, S, and G2/M (mitosis) phases were determined.

### 4.8. Apoptosis Assay

The apoptosis of oral cancer cells was determined by a PE Annexin V Apoptosis Detection Kit with 7-AAD (BioLegend, San Diego, CA, USA) according to the manufacturer’s instructions. Briefly, HSC-3, SAS, and OECM-1 cells (1 × 10^5^) were treated with 0, 10 and 20 mg/mL concentrations of AGA for 72 h. Thereafter, cells were harvested and stained with PE Annexin V/7-AAD for 15 min. The stained cells were analyzed using FACSCanto II low cytometer (BD Biosciences, Franklin Lakes, NJ, USA) and FCS Express software (De Novo, Glendale, CA, USA).

### 4.9. RNA Extraction and Quantitative Real Time-PCR (qRT-PCR)

For qRT-PCR analysis, total RNA was extracted using the PureLink RNA Mini Kit (Invitrogen, Waltham, MA, USA) according to the manufacturer’s instructions. Reverse transcription (RT) was performed as previously described [40]. qRT-PCR was performed using an ABI 7300 real-time PCR system (Applied Biosystems, Foster City, CA, USA), and gene expression was calculated using the 2^−∆Ct^ or 2^−∆∆Ct^ methods with calibration samples included in each experiment.

The primers used were as follows:

- β-actin-forward: 5′–AGAGCTACGAGCTGCCTGAC–3′
- β-actin-reverse: 5′–AGCACTGTGTTGGCGTACAG–3′
- EpCAM- forward: 5′– GCAGCTCAGGAAGAATGTG–3′
- EpCAM–reverse: 5′– CAGCCAGCTTTGAGCAAATGAC–3′
- Survivin-1-forward: 5′–AGGACCACCGACATGTCTACCT–3′
- Survivin-1-reverse: 5′–AAGTCTGGCTCGTTCTCAGTG–3′
- Ki67-forward: 5′ –GCCTGCTCGACCCTACAGA–3′
- Ki67-reverse: 5′–GCTTGTCAACTGCGGTTGC–3′
- PCNA-forward: 5′–CCTGCTGGGATATTAGCTCCA–3′
- PCNA-reverse: 5′–GCGGTAGGTGTCGAAGC–3′
- Bcl2-forward: 5′–CGGAGGCTGGGATGCCTTTG –3′
- Bcl2-reverse: 5′–TTTGGGGCAGGCATGTTGAC–3′
- Bax-forward: 5′-GCCCTTTTGCTTCAGGGTTT–3′
- Bax-reverse: 5′-TCCAATGTCCAGCCCATGAT–3′
- CDK4-forward: 5′– AAATCTTTGACCTGATTGGG–3′
- CDK4-reverse: 5′–CCTTATGTAGATAAGAGTGCTG–3′
- CDK6-forward: 5′–CTGAATGCTCTTGCTCCTTT–3′
- CDK6-reverse: 5′–AAAGTTTTGGTGGTCCTTGA–3′

### 4.10. Western Blot Analysis

Protein extraction and immunoblotting were performed as previously described [41]. The following antibodies were used: cyclin A (BF683) mouse mAb (Cell Signaling Technology #4656, 1:1000), cyclin B1 (D5C10) rabbit mAb (Cell Signaling Technology #12231, 1:1000), cyclin E2 rabbit mAb (Cell Signaling Technology #4132, 1:1000), cyclin D1 (92G2) rabbit mAb (Cell Signaling Technology #2978, 1:1000), vimentin (D21H3) rabbit mAb (Cell Signaling Technology #5741, 1:1000), N-cadherin (4R1H) rabbit mAb (Cell Signaling Technology #13116, 1:1000), β-catenin B (D10A8) rabbit mAb (Cell Signaling Technology #8480, 1:1000) and β-actin (Millipore #MAB1501, 1:10000).

### 4.11. Animal Studies

All the animal studies were approved by The Institutional Animal Care and Use Committee (IUCAC) of Taipei Medical University (Approval no. LAC-2019-0307). Immunodeficiency (NOD/SCID) mice (6 weeks) were purchased from BioLAS Co., Taipei, Taiwan. The animals were housed under pathogen-free conditions and fed with autoclaved food and water. To examine the tumorigenicity in vivo, 2 × 10^6^ SAS cells were subcutaneously injected to induce oral cancer for 7 days. After xenografts reached volumes of 100 mm^3^, the therapeutic AGA extract (500 µL) were administered by oral gavage. The tumor volume was determined by using following formula:

Volume = Length × Width^2^/2

Furthermore, studies on the liver and kidney toxicity of AGA was conducted by morphological observations of hematoxylin and eosin-stained hepatic and renal tissue slices. These results were further corroborated by the blood serum levels of glutamate oxaloacetate (GOT) and glutamate pyruvate transaminase (GPT) representing liver function, whereas blood urea nitrogen (BUN) and serum creatinine indicated renal function.

### 4.12. Statistical Analysis

The sample sizes of all the data are at least *n* = 5, unless otherwise indicated. The data presented are representative of at least three independent experiments. Statistical analyses were performed using GraphPad Prism 5.

## 5. Conclusions

**Figure 7:**
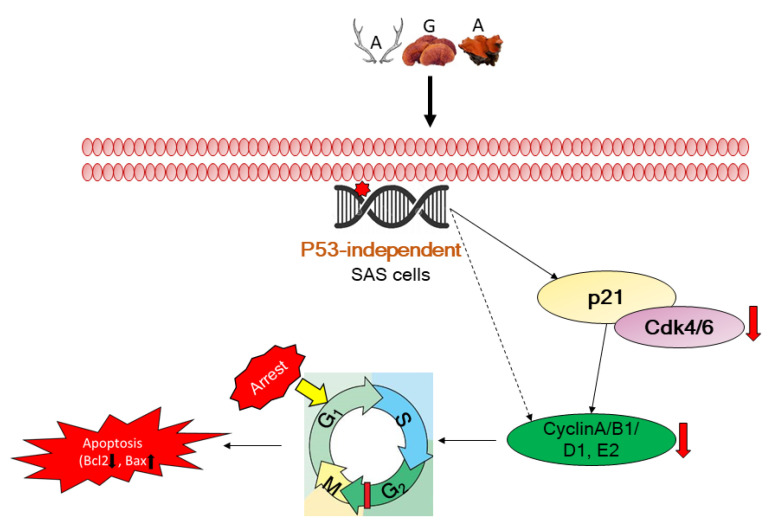
Schematic of possible mechanistic insight of therapeutic action by AGA in oral cancer.

Conclusively, our results revealed the possible therapeutic mechanism associated with AGA therapy in oral cancer, which could be mediated via the inhibition of cancer cell growth and proliferation through enhanced apoptosis and reduced migration (Figure 7).
